# Chemical Oxygen Demand: A Key Determinant in Shaping Biological Community Structure

**DOI:** 10.3390/biology15050418

**Published:** 2026-03-04

**Authors:** Yao Li, Fanqing Kong, Xushen Zhou

**Affiliations:** Center of Eco-Environmental Monitoring and Scientific Research, Administration of Ecology and Environment of Haihe River Basin and Beihai Sea Area, Ministry of Ecology and Environment of the People’s Republic of China, Tianjin 300170, China

**Keywords:** biodiversity, plankton, macrobenthos, nekton, Liaodong Bay

## Abstract

In response to growing global concerns about the impacts of environmental changes on marine ecosystems, scientists have increasingly turned their attention to the role of aquatic environments in shaping biodiversity. This study aimed to assess the biodiversity of northern Liaodong Bay in the context of environmental changes and to elucidate the mechanisms by which aquatic environmental factors influenced different biological groups. Based on the 2024 survey data of phytoplankton, zooplankton, macrobenthos and nekton, the average Marine Biodiversity Index (*MBI*, a comprehensive index) was calculated as 53.08, corresponding to a moderate evaluation level. This suggests a relatively rich diversity of marine species and a fairly even distribution. A correlation analysis between water quality factors and biological community structure reveals that biodiversity in the bay is under pressure from multiple environmental stressors, including elevated COD and heavy metal contamination (e.g., Pb). The study recommends targeted biodiversity conservation strategies and ecosystem management measures to enhance the resilience of the bay’s ecosystem and mitigate the effects of these environmental stressors.

## Simple Summary

This study, based on data collected from the northern Liaodong Bay in August 2024, mainly explores the relationship between marine communities and environmental factors. The results indicate that the region supports relatively high marine species diversity and a fairly even distribution. However, it is under significant pressure from various environmental changes, including elevated levels of chemical oxygen demand (COD) and heavy metal contamination. This study proposes targeted biodiversity conservation and ecosystem management strategies to improve the resilience of the bay and mitigate the impacts of these environmental stressors.

## 1. Introduction

In the context of global environmental changes, biodiversity is facing unprecedented challenges. Rising temperatures, shifts in precipitation patterns, and sea level rise induced by climate change, alongside pollution, habitat destruction, and overfishing driven by anthropogenic activities, have profoundly impacted the structure and functioning of ecosystems [1]. As a critical component of ecosystems, biodiversity is not only the foundation for their stable operation but also a vital resource for human survival [2]. High biodiversity can enhance an ecosystem’s resilience—its ability to withstand and recover from environmental changes—ensuring that ecosystems continue to provide essential services such as food production, climate regulation, and water conservation [3].

The Liaodong Bay ecosystem, a transitional zone between land and sea, is particularly unique in its ecological characteristics and exceptional biodiversity, including diverse habitats, abundant marine flora and fauna, and migration pathways for birds and marine organisms. However, the region is highly vulnerable to environmental changes and is among the most severely affected marine areas due to anthropogenic activities [4]. As urban areas expand rapidly and agricultural practices intensify, rivers flowing into Liaodong Bay transport a wide array of pollutants from both anthropogenic and natural sources. The Liao River, Daliao River, Xiaoling River, and Daling River are the primary contributors [5], with pollutants including nutrients (N and P), COD, heavy metals, trace organic pollutants, and petroleum hydrocarbons. Additionally, atmospheric deposition has further increased nutrient input, exacerbating nutrient concentrations in the bay [6]. Despite a reduction in the concentrations of dissolved inorganic nitrogen (DIN) and dissolved inorganic phosphorus (DIP) in Bohai Bay between 2006 and 2020, eutrophication remains a persistent issue [7]. The total volume of terrestrial nutrients entering the bay continues to rise, dramatically altering nutrient dynamics and distribution patterns. Simultaneously, the benthic dissolved oxygen (DO) concentration has decreased steadily in summer [8], posing a serious threat to the stability and biodiversity of the marine ecosystem [9].

Environmental changes have multi-dimensional effects on the bay ecosystem. Warming seawater due to climate change may disrupt the structure of plankton communities, leading to the decline of species adapted to cooler environments and increases in heat-tolerant species [10]. Rising sea levels will inundate critical habitats, such as coastal wetlands and mangroves, adversely affecting benthic organisms and nekton dependent on these ecosystems [11]. Furthermore, anthropogenic pollution—such as nutrient-induced red tides and the toxic effects of heavy metals on organisms—significantly threatens the bay ecosystem’s biodiversity and ecological functions [12]. Despite this, research on the combined effects of multiple environmental changes on biodiversity remains relatively limited. In particular, in-depth studies in specific regions remain insufficient. Therefore, given that Liaodong Bay is under environmental pressure, comprehensive research on the bay’s biodiversity under environmental stressors—along with the development of effective protection and management strategies—is crucial for maintaining the health and stability of the bay ecosystem.

## 2. Materials and Methods

### 2.1. Overview of the Research Area

Liaodong Bay is a semi-closed bay surrounded by diverse ecosystems, including land, estuaries, and shallow seas. The annual average temperature ranges from 7 °C to 9.5 °C, with relatively higher temperatures in coastal areas, plains, and hills, and cooler temperatures in the eastern mountains [5]. Annual precipitation is between 670 and 800 mm, which is moderate. The bay is surrounded by industrial parks and residential areas, where frequent activities such as industrial wastewater discharge, domestic sewage disposal, port shipping, and fishing have affected the local ecology [9]. Additionally, global climate change is causing rising temperatures, sea levels, and shifts in precipitation patterns in the region.

### 2.2. Sampling Methods

#### 2.2.1. Phytoplankton Sampling

**Figure 1:**
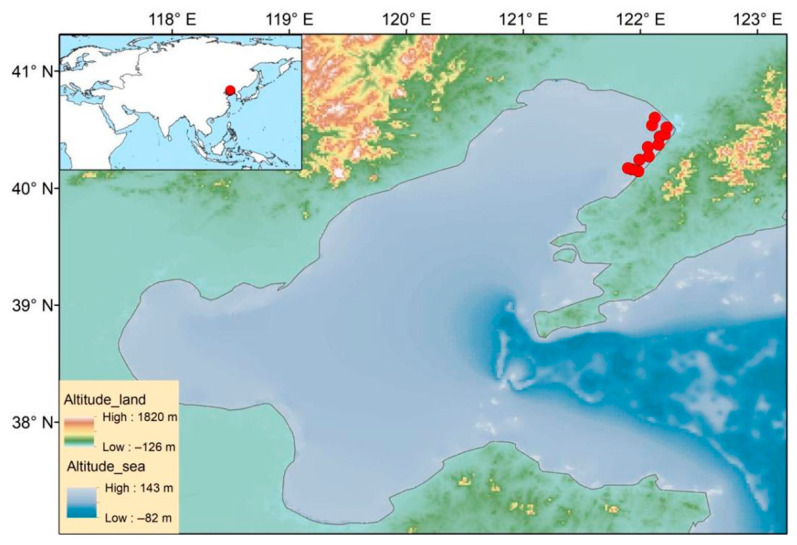
Map of the research area.

The average water depth in the research area is about 7 m. In August 2024, twelve sampling sites were set up in the bay (Figure 1), and a shallow water Type III plankton net (GuangZhou HaiKangOcean Technology Co., Ltd., Guangzhou, China. Equipment in Section 2.2.2, Section 2.2.3 and Section 2.2.4 are from the same manufacturer.) was used for vertical trawling to collect phytoplankton samples. The samples were immediately fixed with Lugol’s solution. In the laboratory, they underwent treatments such as precipitation and concentration. Species identification and cell counting were performed under a microscope, with species determined based on the relevant taxonomic literature [13]. (The Type III plankton net, with a net mouth area of 0.1 m^2^, is suitable for the vertical or segmented collection of small plankton in shallow waters within 30 m).

For relevant information on Section 2.2.1, Section 2.2.2, Section 2.2.3 and Section 2.2.4, refer to GB/T 12763.6-2007 [14].

#### 2.2.2. Zooplankton Sampling

In August 2024, zooplankton samples were also collected at the same 12 sampling sites using shallow water Type I and Type II plankton nets for vertical trawling. The sampling time was the same as for phytoplankton. The samples were immediately fixed with Lugol’s solution, sorted, identified, and counted in the laboratory. Species were identified based on their morphological characteristics and taxonomic data [15]. The Type I plankton net, with a net mouth area of 0.2 m^2^, is suitable for the vertical or segmented collection of large and medium-sized zooplankton, as well as fish larvae. The Type II plankton net, with a net mouth area of 0.08 m^2^, is suitable for the vertical or segmented collection of small and medium-sized zooplankton, as well as dinoflagellates (in waters ≤ 30 m).

#### 2.2.3. Macrobenthos Sampling

In August 2024, macrobenthos were collected from the same 12 sampling sites using a grab sediment sampler with a sampling area of 0.125 m^2^ and a sediment sampling depth of 10 cm. The sediment samples were then sieved through a 0.5 mm mesh, and the benthic organisms were picked out. To facilitate species identification and biomass measurement, the samples were preserved in 75% ethanol, with species identification based on macrobenthos classification atlases and relevant literature [16].

#### 2.2.4. Nekton Sampling

In August 2024, nekton sampling was conducted using an Agassiz trawl (GuangZhou HaiKangOcean Technology Co., Ltd., Guangzhou, China). Each trawl lasted 1 h, with a net speed of approximately 3 knots. One trawl was conducted at each of the 12 sampling sites. The specifications of the trawl used for nekton sampling are as follows: mouth width 3.0 m, height 0.8 m, body mesh size 5 mm and cod-end mesh size 1 mm. The deployment depth range for the trawl was between 0 and 5 m. Captured nekton samples were identified, counted, and weighed on-site. For species that were difficult to identify immediately, photos were taken for later identification in the laboratory. Species classification was carried out using relevant literature on the taxonomy of fish, shrimp, crabs, and cephalopods [17,18].

### 2.3. Environmental Data Collection

Meteorological data for the study area, including temperature, N, P, DO, etc., were collected from local marine environmental monitoring stations. The data collection and analysis strictly followed the national standard for marine water quality (GB 3097-2017) [19].

### 2.4. Data Analysis Methods

Sampling site distribution and study area maps were produced using ArcGIS Desktop 10.8.2 (Esri Inc., Redlands, CA, USA). The topographic and bathymetric data were obtained from the ETOPO1 Bedrock global relief model, and the land–sea boundary was derived from the built-in geographic database of ArcMap (Esri Inc., Redlands, CA, USA). Both datasets are publicly available for academic use.

The PRIMER 6 software was used for the initial organization and statistical analysis of biodiversity data, as well as to calculate various biodiversity indices, such as the Shannon–Wiener Index (*H*′), Pielou’s evenness index (*J*′), Margalef’s richness index (*d*), the Index of Relative Importance (*IRI*) for nekton, etc.

AMBI (AZTI’s Marine Biotic Index) classifies benthic organisms into five ecological groups based on their varying degrees of sensitivity to environmental pollution, specifically organic matter enrichment: Group I (G I) includes species that are very sensitive to organic enrichment and thrive under unpolluted conditions; Group II (G II) consists of species that are not particularly sensitive to organic enrichment; Group III (G III) includes species that can tolerate high levels of organic matter enrichment; Group IV (G IV) represents secondary opportunistic species, which thrive in conditions ranging from slight to severe imbalance; and Group V (G V) comprises primary opportunistic species, found in conditions of severe imbalance. The calculation formula is as follows:

AMBI = [(0 × %GI) + (1.5 × %GII) + (3 × %GIII) + (4.5 × %GIV) + (6 × %GV)]/100

The AMBI index has been widely applied and validated in coastal ecosystems, including Liaodong Bay, demonstrating good applicability in this region. Region-specific species ecological-group assignment was performed according to the ecological traits of macrobenthos in Liaodong Bay to ensure the reliability of the assessment results. AMBI was calculated using AMBI V5.0 software.

The *MBI* calculation formula is as follows:

$$MBI=\sum_{i=1}^{5}W_{i}\sum_{j=1}Y_{ij}W_{ij}$$

*MBI*—comprehensive index of marine biodiversity.

*W_i_*—weight of the *i*-th primary indicator.

*Y_ij_*—assigned score of the *j*-th secondary indicator under the *i*-th primary indicator.

*W_ij_*—weight of the *j*-th secondary indicator under the *i*-th primary indicator. Equal weights are adopted for the evaluation indicators, and the sum of weights of indicators at each level is equal to 1.

For relevant information on AMBI and *MBI*, refer to HY/T 215-2017 [20].

The single pollutant factor evaluation method is used to assess water quality (such as COD, DIN, PO_4_^3−^ and other key parameters).

A Pearson correlation analysis was conducted using Python 3.9.4 [21], and a redundancy analysis (RDA) was performed using Canoco 5.0 software to identify key environmental factors influencing biological diversity. Before the statistical analysis, the normality of the environmental factor data was tested using the Shapiro–Wilk test (implemented in Scipy 1.10.1), and the homogeneity of variances was verified with Levene’s test (implemented in the Statsmodels package, version 0.14.0). To avoid multicollinearity among environmental variables, for variable pairs with a correlation coefficient of |r| ≥ 0.7, the variable with stronger ecological relevance to the biological community was retained. Multicollinearity was further evaluated using variance inflation factor (VIF) analysis, which was performed in Python 3.9.2 with the statsmodels 0.14.0 package; all VIF values were <3, confirming no significant multicollinearity. Prior to conducting the RDA, the species density dataset were subjected to log_10_(*x* + 1) transformation. This transformation aimed to reduce the overwhelming influence of extremely high values on the ordination results, stabilize the variance of non-normally distributed biological data, and balance the numerical scales among taxa with distinct magnitude differences. The significance of the ordination model and individual environmental factors was tested using Monte Carlo permutation tests (999 permutations) within Canoco 5.0.

## 3. Results

**Figure 2:**
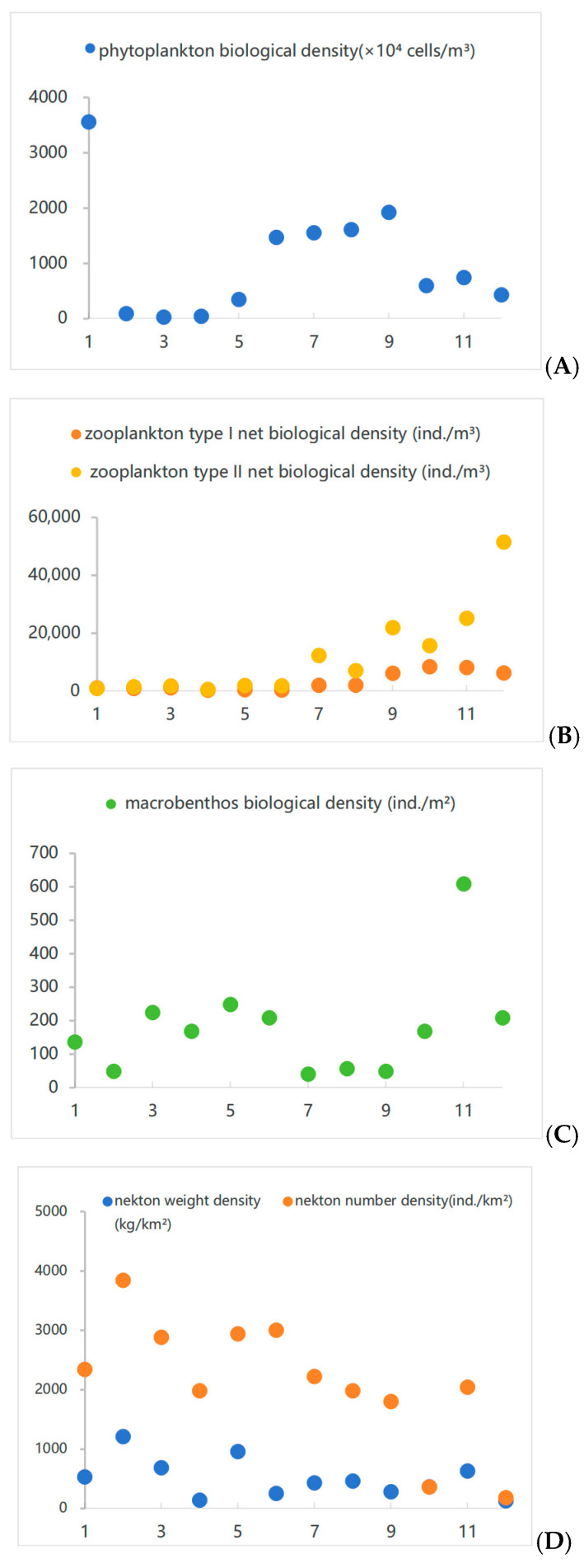
Density of various biological groups. ((**A**–**D**) represent the density of phytoplankton, zooplankton, macrobenthos, and nekton, respectively).

**Figure 3:**
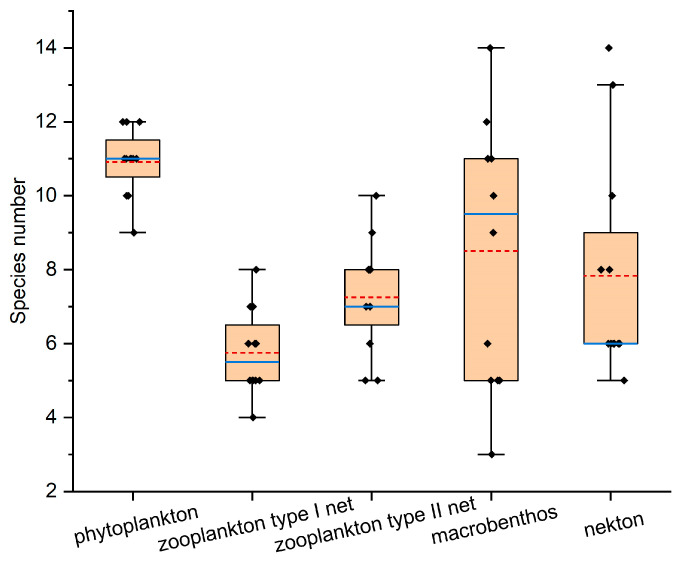
The number of species in each biological group. (The solid blue lines represent the median, and the dashed red lines represent the mean. The box limits are 25% and 75%. The whiskers represent 1.5 times the interquartile range).

**Figure 4:**
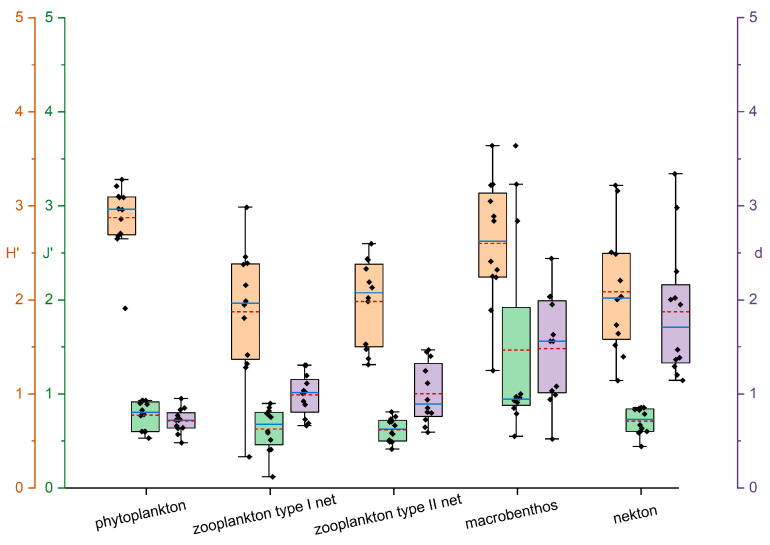
Diversity indices of different biological groups. (Y-axis colors: orange represents *H*′, green represents *J*′, and purple represents *d*. The solid blue lines represent the median, and the dashed red lines represent the mean. The box limits are 25% and 75%. The whiskers represent 1.5 times the interquartile range).

The results from Figure 2, Figure 3 and Figure 4 showed that a total of 35 phytoplankton species were identified in northern Liaodong Bay in August 2024, belonging to three phyla: Cyanophyta, Bacillariophyta, and Pyrrophyta. Among them, 31 species were Bacillariophyta (88.6%), 3 species were Pyrrophyta (8.6%), and 1 species was Cyanophyta (2.8%). The cell density ranged from 2.8 × 10^5^ to 3.6 × 10^7^ cells/m^3^, with an average of 1.0 × 10^7^ cells/m^3^. The dominant species were *Pseudo-nitzschia pungens* (dominance = 0.16), *Skeletonema costatum* (dominance = 0.15), and *Coscinodiscus wailesii* (dominance = 0.10). The community structure of phytoplankton was analyzed using *H*′ and *J*′. The *H*′ values ranged from 2.51 to 3.13, indicating a slight level of organic pollution [22], *J*′ values from 0.74 to 0.91, and *d* values from 0.60 to 0.69. In areas strongly influenced by environmental changes, both *H*′ and *J*′ values were lower, suggesting reduced stability and species diversity in the phytoplankton community.

Twenty-one zooplankton species were identified, belonging to four phyla: Arthropoda, Chaetognatha, Urochordata, and Cnidaria. Arthropoda had the highest number of species (11 species, 52.38% of the total), followed by Cnidaria (7 species, 33.33%). The average zooplankton abundance was 15,159.25 ind./m^3^. There are three main dominant species: *Aidanosagitta crassa* (dominance = 0.29), *Labidocera euchaeta* (dominance = 0.28), and *Acartia pacifica* (dominance = 0.20). The zooplankton abundance ranged from 720.36 to 57,660.00 ind./m^3^. The *H*′, *J*′, *d* ranged from 1.69–2.44, 0.46–0.75 and 1.08–1.35, respectively.

Sixty-eight macrobenthic species were identified across eight phyla: Arthropoda, Mollusca, Annelida, Nemertea, Echiura, Brachiopoda, Echinodermata, and Cnidaria. Annelida had the highest species count (31 species, 45.59%), followed by Arthropoda (16 species, 23.53%) and Mollusca (14 species, 20.59%). There were two main dominant species: *Amphioplus japonicus* (dominance = 0.02) and *Ampelisca bocki* (dominance = 0.03). The macrobenthic biomass ranged from 0.32 to 1400.89 g/m^2^, with an average of 120.6 g/m^2^. The density ranged from 8 to 608 ind./m^2^, with an average of 166.8 ind./m^2^. The *H*′, *J*′, *d* fluctuated of 1.94–3.24, 0.8–0.92 and 0.86–2.01, respectively.

Twenty nekton species were identified, representing 16 families and 8 genera. Among these, 13 were fish (65%), 3 were shrimp (15%), 3 were crabs (15%), and 1 was a cephalopod (5%). The average biomass for fish was 179.25 kg/km^2^, that for crustaceans was 236.67 kg/km^2^, and that for cephalopods was 6.81 kg/km^2^. The four main dominant species (*IRI* > 1000) were *Charybdis japonica*, *Mugil cephalus*, *Oratosquilla oratoria*, and *Acanthopagrus schlegelii*. The nekton catch varied from 55.99 to 549.44 kg/km^2^ for fish, from 7.45 to 1128.02 kg/km^2^ for crustaceans, and from 6.14 to 29.06 kg/km^2^ for cephalopods. The *H*′, *J*′, and *d* ranged from 1.46–2.56 to 0.59–0.82 and 1.29–2.32, respectively.

The marine biodiversity of the northern part of Liaodong Bay was assessed with an average *MBI* of 53.08, indicating medium biodiversity (Table 1). This suggests a relatively rich variety of marine species, with fairly even distribution. Some local areas and biological communities show particularly high species diversity, and certain ecosystems are highly enriched.

**Figure 5:**
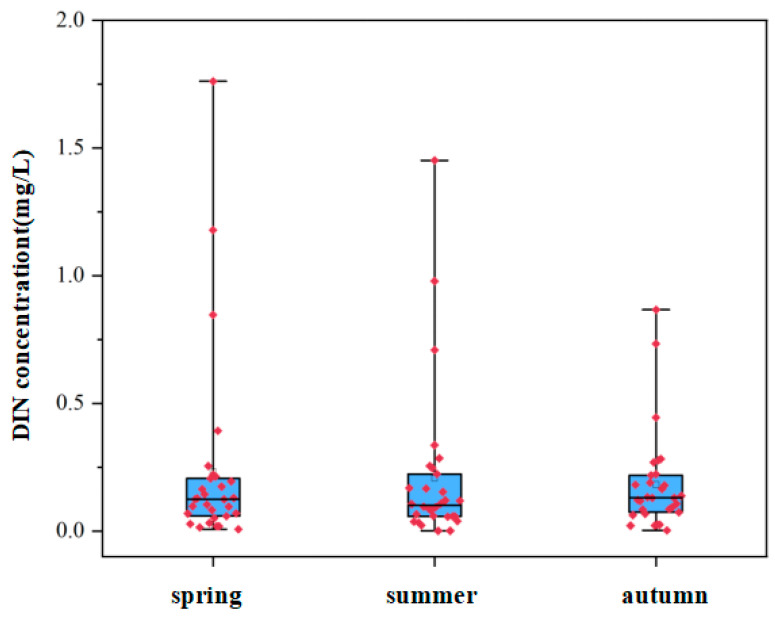
The variation range of the DIN concentration in seawater quality in 2024. (The solid black lines represent the median. The box limits are 25% and 75%. The whiskers represent 1.5 times the interquartile range).

The selected environmental data are spatially consistent with the biological data, and time-series data from the same season as the biological sampling were used for the subsequent analysis. The water quality data collected in 2024 showed that, in certain areas of northern Liaodong Bay, the proportion of seawater meeting the excellent water quality standard was below 85% [19]. The primary pollutants affecting seawater quality are DIN and PO_4_^3−^. Figure 5 shows that the maximum concentration of DIN in summer is 1.452 mg/L. Therefore, eutrophication ranging from mild to severe is occurring in different regions.

**Figure 6:**
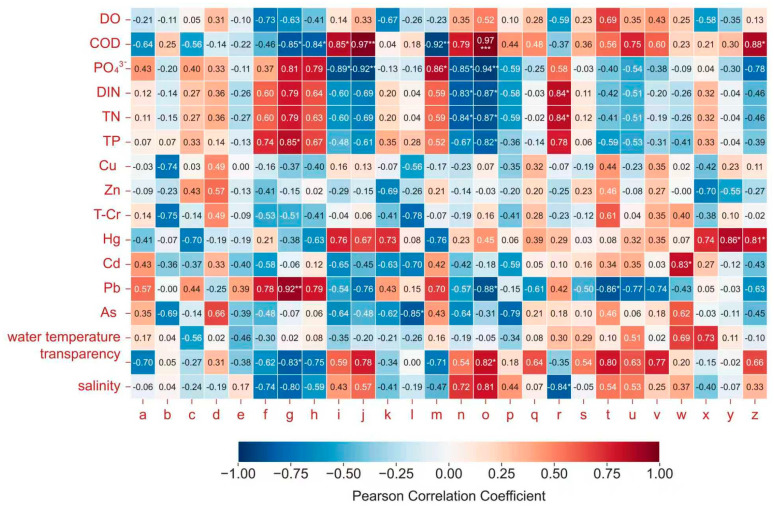
Correlation heatmap of marine biological communities and water quality factors. (Note: 1. a~z, respectively, represent the number of phytoplankton species, the phytoplankton cell density, phytoplankton *H*′, phytoplankton *J*′, phytoplankton *d*, the number of species in Type I nets of zooplankton, zooplankton Type I net abundance, the biomass of zooplankton Type I nets, zooplankton Type I net *H*′, zooplankton Type I net *J*′, zooplankton Type I net *d*, the number of species in Type II nets of zooplankton, the zooplankton Type II net abundance, zooplankton Type II net *H*′, zooplankton Type II net *J*′, zooplankton Type II net *d*, the number of macrobenthos, the density of macrobenthos, the biomass of macrobenthos, macrobenthos *H*′, macrobenthos *J*′, macrobenthos *d*, macrobenthos AMBI, the number of nekton, the weight density of nekton, and the number density of nekton. 2. The significance asterisks marked in the correlation heat map are defined as: * *p* < 0.05 and ≥0.01, ** *p* < 0.01 and ≥0.001, *** *p* < 0.001. No marking indicates no statistical significance (*p* ≥ 0.05). 3. No multiple comparison correction was performed for the correlation analysis results).

**Figure 7:**
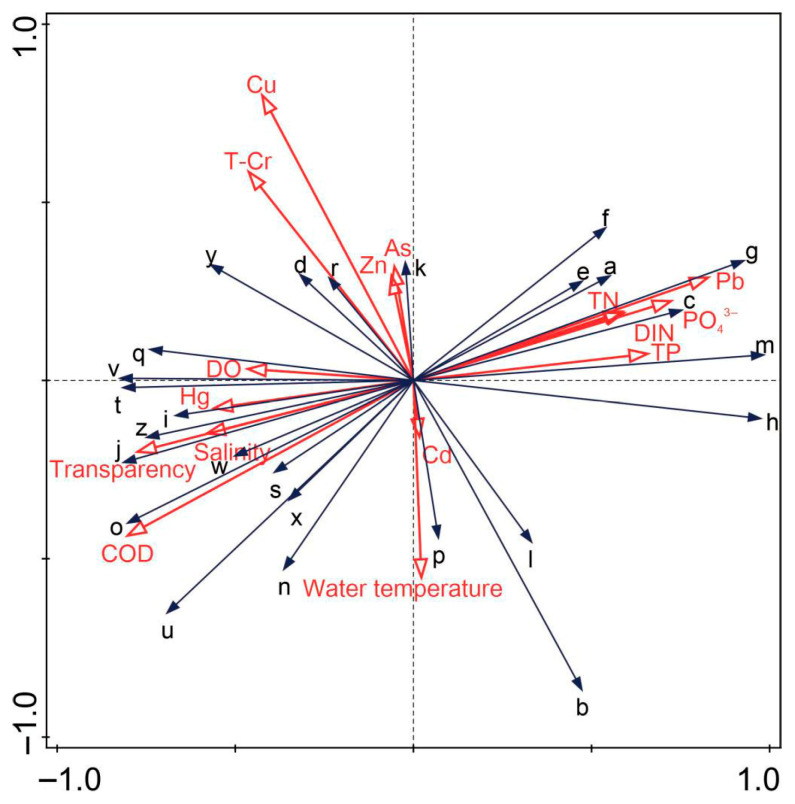
RDA of biological communities and environmental factors. (RDA1 and RDA 2 account for (59.3%) and (20.4%) of the total variance, respectively. The red arrows indicate environmental variables, and the black arrows a–z have the same meaning as in Figure 6; the length of each arrow represents the strength of the correlation between the environmental variable and the community structure, while the direction of the arrow indicates the gradient of the environmental variable).

Pearson correlation analysis was conducted to quantify the linear relationships between individual environmental factors and specific biological indices, while RDA was applied to clarify the overall effects of the combined environmental variables on the biological community structure (Figure 6 and Figure 7). The results of the Shapiro–Wilk test and Levene’s test indicated that all datasets met the assumptions for parametric tests (*p* > 0.05); therefore, no data transformation (e.g., log_10_(*x* + 1)) was applied in the Pearson correlation analysis.

Prior to RDA, the species density dataset was subjected to log_10_(*x* + 1) transformation. The final set of environmental variables used in RDA included heavy metals, temperature, salinity, COD, DIN, PO_4_^3−^ and other key parameters. RDA was accompanied by Monte Carlo permutation tests (999 permutations) to verify model significance (*p* < 0.05, F = 3.1). The first two canonical axes (RDA1 and RDA2) explained 59.3% and 20.4% of the variance in the species data constrained by environmental variables, respectively, with a cumulative explained variance of 79.7%.

As illustrated in Figure 6 and Figure 7, Pearson correlation analysis conducted with Python reveals that COD, Pb, PO_4_^3−^ and DIN significantly influence biological community structure and characteristics (*p* < 0.05). In particular, COD was significantly positively correlated with the zooplankton *H*′ and *J*′ (r = 0.97, *p* < 0.001); Pb was significantly positively correlated with the species count, abundance and biomass of Type I zooplankton ($\bar{r}$ = 0.83, *p* < 0.01); nutrients (PO_4_^3−^ and DIN) were significantly positively correlated with the density of Type II zooplankton and macrobenthos (r = 0.86, *p* < 0.05 and r = 0.84, *p* < 0.05, respectively).

## 4. Discussion

Coastal areas of China are experiencing widespread nutrient and trace metal contamination, primarily driven by anthropogenic activities [23,24]. In the present study, although dissolved heavy metal concentrations in Liaodong Bay did not exceed Class I water quality standards, relatively elevated levels were still observed, which were closely related to river runoff and suspended sediment input, indicating persistent anthropogenic pressures [25,26,27,28,29]. These findings highlight the importance of long-term monitoring; even low metal concentrations may pose potential risks to coastal ecosystems. Our results further confirm that water quality indicators such as dissolved oxygen and COD can effectively reflect anthropogenic disturbances, consistent with previous observations [28], which underscores the necessity of targeted nutrient concentration management and adaptive ecosystem protection strategies [29].

(I) Plankton

As sensitive primary producers and consumers with rapid turnover rates, plankton communities strongly reflect environmental conditions and water quality changes [30,31].

In this study, phytoplankton cell density in northern Liaodong Bay exhibited obvious spatial and temporal heterogeneity: higher values occurred nearshore and within estuarine zones, reflecting terrestrial nutrient subsidies, while peaks in August corresponded to favorable temperature, light, and nutrient availability. Zooplankton abundance followed similar spatiotemporal patterns, likely driven by bottom-up control from phytoplankton food availability [32,33].

Studies have shown that temperature, salinity, nitrite, and COD affect the density, population structure, and species composition of zooplankton communities [34,35,36]. Salinity, suspended particulate matter, water and sediment pH, COD, and chlorophyll a have the most significant impacts on zooplankton biomass. The abundance and biomass of zooplankton are shaped by the combined effects of environmental and biological factors [37,38]. According to the Pearson correlation and RDA results in this study, COD was identified as the primary environmental driver shaping the overall biological community structure, followed by PO_4_^3−^ and DIN. These nutrients directly regulate phytoplankton growth, species composition, and diversity. While balanced nutrient levels support marine biodiversity, excessive nutrients can cause eutrophication, leading to rapid phytoplankton growth, dissolved oxygen depletion, and harmful algal blooms that threaten fish and benthic organisms [39]. Intensified anthropogenic activities have disturbed the N/P ratio and exacerbated hypoxia in semi-enclosed bays such as Liaodong Bay, where low flushing rates accelerate nutrient accumulation [40].

Heavy metals (Cu, Pb, Zn, Cd) also affect the plankton community structure. The effects of heavy metals on phytoplankton are dual and non-linear: low concentrations of essential metals (Cu, Zn) may promote growth, while high concentrations and non-essential metals (Pb, Cd) inhibit growth and alter community structure [41,42,43,44,45,46]. Phytoplankton at low trophic levels are more sensitive to heavy metals due to their strong bioaccumulation capacity [47]. Heavy metal stress tends to reduce overall diversity by eliminating sensitive taxa while favoring tolerant species, and may directly impair photosynthesis [48,49,50,51,52,53,54,55].

(II) Macrobenthos and Nekton

Macrobenthos are ideal bioindicators for assessing benthic environmental quality due to their limited mobility and high sensitivity to environmental disturbances [56,57,58]. Marine species such as fish, invertebrates, and aquatic plants can indicate ecological disturbances and contamination levels [59,60].

In this study, the average AMBI value was 1.71, indicating mild disturbance and moderate overall benthic ecological quality. Macrobenthos biomass and density were consistently higher in areas with good sediment quality and stable hydrological conditions, but decreased markedly in heavily disturbed zones, consistent with the AMBI-derived disturbance gradient. For nekton, overfishing and environmental changes have shifted the community toward small-sized fish and juveniles, reducing diversity and stability. Overfishing may have exacerbated this community shift, as the removal of predators has potentially weakened the top-down control of dominant invertebrates [61,62,63].

Different from plankton, macrobenthos were more strongly affected by PO_4_^3−^ and DIN. Elevated NO_2_^−^-N and NO_3_^−^-N can induce nitrogen saturation, interfering with environmental filtering and reducing benthic diversity [64,65,66]. Nutrient enrichment also altered biofilm characteristics and affected invertebrate settlement [67,68,69,70], which collectively determined macrobenthic community structure in Liaodong Bay [71,72].

At higher trophic levels, fish communities were mainly affected by temperature, salinity, pH, and chlorophyll a [73]. Since benthic invertebrates efficiently bioaccumulate heavy metals, these contaminants can be transferred and biomagnified along the food web, posing potential risks to higher consumers and human health [74].

(III) Biodiversity conservation and recommendations

Spatio-temporal variations in biodiversity indices reveal significant spatial heterogeneity across northern Liaodong Bay [75,76,77]. Our research results indicate that the marine biodiversity in the northern part of Liaodong Bay was assessed with an average *MBI* of 53.08, corresponding to a moderate level of biodiversity. This suggests a relatively rich variety of marine species, with fairly even distribution. Notably, several local habitats and biological communities exhibit exceptionally high species diversity, and certain ecosystem types are characterized by high species enrichment. These quantitative results provide direct evidence that the overall ecological status of Liaodong Bay is improving and the ecosystem is shifting toward greater stability. It should be acknowledged that the present study was limited by the requirements of the cooperative project, with only one field survey implemented. Future research is, therefore, recommended to incorporate multiple seasonal or annual surveys to enhance the reliability and representativeness of the results.

In ecosystems with high species richness, different species can sustain ecosystem functions through functional complementarity and redundancy mechanisms when faced with environmental changes or disturbances [78]. Increased water turbidity and organic matter can reduce native fish and zooplankton populations, alter benthic assemblages, and hinder ecosystem services [79,80]. This decline in water quality and biodiversity may erode ecosystem functions and services [81].

Biodiversity supports ecosystem functioning in natural assemblages, even amid environmental changes [82,83,84]. Species richness boosts community biomass production, a key indicator of ecosystem function, which is crucial for many ecosystem goods and services [82]. Benkwitt [83] found that species richness and functional diversity in both large (fish, macrophytes) and small (microcrustaceans, rotifers, protists, and phytoplankton) aquatic organisms positively correlate with ecosystem multifunctionality. Whereas the positive association between smaller organisms and multifunctionality broke down with increasing human pressure, this positive relationship was maintained for larger organisms despite the increase in human pressure. Recent research has underscored the vital role of biodiversity in maintaining ecosystem resilience [85]. Studies indicate that ecosystems with high levels of biodiversity recover more rapidly after disturbances [86,87].

Against this backdrop, targeted strategies are proposed to enhance marine biodiversity in the nearshore of Liaodong Bay by mitigating the impact of anthropogenic activities, including the following key aspects: (1) strengthen water quality monitoring and targeted pollution control to reduce nutrient loading (DIN, PO_4_^3−^), COD, and organic enrichment; (2) establish a comprehensive biodiversity database to effectively monitor temporal and spatial changes; (3) integrate advanced technologies (e.g., satellite remote sensing, UAV monitoring) into field surveys for continuous and targeted assessments [88]; and (4) strengthen AI-based early warning systems for ecological threats (e.g., red tides, jellyfish blooms) and protect key ecosystems.

## 5. Conclusions

Based on data collected from northern Liaodong Bay in August 2024, this study assessed the biodiversity and environmental conditions of the study area using multiple indices and elucidated the key environmental factors shaping the biological community structure—thus achieving the core research objective of clarifying the biodiversity dynamics and environmental factor regulation mechanisms in the northern Liaodong Bay under ongoing environmental changes. This study yields three primary scientific conclusions with distinct regional novelty and ecological significance: first, the overall ecological status of northern Liaodong Bay is at a moderate level, with mild anthropogenic disturbance and pollution; second, nutrient enrichment and heavy metal stress are the dominant environmental stressors, with COD, Pb, PO_4_^3−^, and DIN identified as the key factors disrupting regional species composition and biological community structure; third, the physicochemical variations of the water column are driven by the combined effects of anthropogenic activities and natural variability, exhibiting both short-term fluctuations and long-term trends that cannot be attributed to a single environmental factor.

Quantitatively, the average *H*′ values for plankton, macrobenthos, and nekton indicate mild pollution. An average AMBI value of 1.71 denotes mild disturbance, with the overall benthic ecological quality classified as moderate. The *MBI* results also reflect a medium status, which is indicative of a relatively rich marine species and fairly even distribution. These findings verify the study’s initial hypothesis that anthropogenically induced environmental stress has not caused severe biodiversity loss in northern Liaodong Bay but has already led to subtle ecological disturbances and community structural imbalances. Notably, the reduced body size of fishery-targeted species and unstable fishery community structure represent key ecological concerns that require targeted management interventions.

Correlation analyses further reveal clear linkages between marine biological communities and environmental factors in the study region. The study area is facing severe nutrient loading pressure, with unstable water quality that frequently fails to meet the national marine environmental quality standards. Both anthropogenic activities and natural variability have caused asynchronous fluctuations in nutrient concentrations, thereby negatively impacting ecological balance. Consistent with the above conclusions, key environmental factors such as COD, Pb, PO_4_^3−^, and DIN have disrupted species composition and biological community structure.

In the context of climate change, the physicochemical variations in the water column involve both short-term fluctuations and long-term trends, further highlighting the complexity of coastal environmental changes that cannot be attributed to a single factor. Future research should expand the spatial and temporal scope of field investigations to better clarify the long-term coupling relationship between biodiversity and environmental changes in northern Liaodong Bay and to develop scientific and effective ecological strategies for addressing and mitigating emerging ecological challenges in this typical semi-enclosed bay of the Bohai Sea.

## Figures and Tables

**Table 1:** Classification of marine biodiversity status.

*MBI* Level	*MBI*	The Current Situation of *MBI*
High	≥75–100	High species richness, even distribution; high diversity across communities; diverse ecosystem types.
Medium	≥50–<75	Moderate species richness, even distribution; high diversity in local communities/regions; rich ecosystems in partial areas.
General	≥25–<50	Limited species richness, uneven distribution; local high diversity; overall moderate biodiversity.
Low	0–<25	Scarce species, uneven distribution; simple ecosystem types; low overall biodiversity.

## Data Availability

The data presented in this study are available upon request from the corresponding author. Due to the fact that the data are currently confidential as required by national regulations, relevant information will be shared publicly once declassified.

## References

[1] IPCC (2023). Climate Change 2023: Synthesis Report.

[2] Whitesel T.A., Sankovich P.M. (2025). Climate Projections and Pacific Lamprey Conservation: Evidence That Larvae in Natural Conditions May Be Resilient to Climate Warming. Biology.

[3] Folke C., Carpenter S.R., Walker B., Scheffer M., Elmqvist T., Gunderson L.H., Holling C.S. (2004). Regime Shifts, Resilience, and Biodiversity in Ecosystem Management. Annu. Rev. Ecol. Evol. Syst..

[4] Lotze H.K., Lenihan H.S., Bourque B.J., Bradbury R.H., Cooke R.G., Kay M.C., Kidwell S.M., Kirby M.X., Peterson C.H., Jackson J.B. (2006). Depletion, degradation, and recovery potential of estuaries and coastal seas. Science.

[5] Wu G., Qiu M., Li J., Luo W. (2023). Spatial-temporal variation of water quality and pollutant source analysis in rivers along Liaodong Bay. Acta Oceanol. Sin..

[6] Zheng L., Zhai W. (2023). Nutrient dynamics in the Bohai and North Yellow seas from seasonal to decadal scales: Unveiling Bohai Sea eutrophication mitigation in the 2010s. Sci. Total Environ..

[7] Zhang X., Tian C., Sun Z., Yin X., Sun R., Wang J. (2024). Temporal and Spatial Distribution of DIN and DIP Concentrations and Source Apportionment Along the Bohai Sea of China During 2015–2022. Chin. Geogr. Sci..

[8] Qu L.M., Feng P.F., Cong P.F., Tao G.F., Liu H.Y., Duan W.Y. (2025). Spatial-temporal distribution of nitrogen and phosphorus nutrients and potential eutrophication assessment in Bohai Bay, China. Ocean Coast. Manag..

[9] Ning X.R., Li D.Q., Wang Y.S., Zhang J. (2010). Long-Term Environmental Changes and the Responses of the Ecosystems in the Bohai Sea During 1960–1996. Deep Sea Res. Part II Top. Stud. Oceanogr..

[10] Beaugrand G., Reid P., Ibañez F., Lindley J., Edwards M. (2002). Reorganization of North Atlantic Marine Copepod Biodiversity and Climate. Science.

[11] Barbier E.B., Koch E.W., Silliman B.R., Hacker S.D., Wolanski E., Primavera J., Granek E.F., Polasky S., Aswani S., Cramer L.A. (2008). Coastal Ecosystem-Based Management with Nonlinear Ecological Functions and Values. Science.

[12] Anderson C.A., Bushman B.J. (2002). Human aggression. Annu. Rev. Psychol..

[13] Wang J. (2003). Species Composition and Quantity Variation of Phytoplankton in Inshore Waters of the Bohai Sea.

[14] (2007). Specifications for Oceanographic Survey—Part 6: Marine Biological Survey. General Administration of Quality Supervision, Inspection and Quarantine of the People’s Republic of China.

[15] Bi H.S., Sun S., Gao S.W. (2001). Ecology of Zooplankton Communities in the Bohai Sea.

[16] Li X.Z. (2022). Atlas of Morphological Classification of Common Benthic Animals in the Bohai Sea.

[17] Liu M., Shao K.Z., Hsueh I.W., Zhang H. (2023). Illustrated Marine Fishes of China.

[18] Institute of Oceanology, Chinese Academy of Sciences (2020). Fauna Sinica (Crustacea, Cephalopoda).

[19] (2017). Seawater Quality Standard.

[20] (2017). Guideline for Marine Biodiversity Assessment in Nearshore Area.

[21] Virtanen P., Gommers R., Oliphant T.E., Haberland M., Reddy T., Cournapeau D., Burovski E., Peterson P., Weckesser W., Bright J. (2020). SciPy 1.0: Fundamental algorithms for scientific computing in Python. Nat. Methods.

[22] Hayes A., Kucera M., Kallel N., Sbaffi L., Rohling E. (2005). Glacial Mediterranean sea surface temperatures based on planktonic foraminiferal assemblages. Quat. Sci. Rev..

[23] Jiang T., Zhang Y.J., Bao Z.F. (2018). Study on Water Level Control for Lake Water Quality Management: A Case Study of Ci Lake. J. Wuhan Univ. (Eng. Ed.).

[24] Thrush S.F., Dayton P.K. (2002). Disturbance to Marine Benthic Habitats by Trawling and Dredging: Implications for Marine Biodiversity. Annu. Rev. Ecol. Evol. Syst..

[25] Collie J.S., Hall S.J., Kaiser M.J., Poiner I.R. (2000). A quantitative analysis of fishing impacts on shelf-sea benthos. J. Anim. Ecol..

[26] Myers R., Worm B. (2003). Rapid worldwide depletion of predatory fish communities. Nature.

[27] Pauly D., Christensen V., Dalsgaard J., Froese R., Torres F. (1998). Fishing Down Marine Food Webs. Science.

[28] Yang G.J., Wu Z.X., Song L., Lu X.Q. (2018). Seasonal variation of environmental variables and phytoplankton community structure and their relationship in Liaodong Bay of Bohai Sea, China. J. Ocean Univ. China.

[29] Diaz R.J., Rosenberg R. (2008). Spreading Dead Zones and Consequences for Marine Ecosystems. Science.

[30] Fott B., Otto Glenk H. (1971). Algenkunde.

[31] Zhang G.Q., Yang Y.L., Tang A.G. (2020). The community structure of Phytoplankton in the Xin’an River Basin (Tunxi Section) and its relationship with Environmental factors. Chin. J. Ecol..

[32] Zhang S., Shi B.J., Xie B., Zhang H., Li D.P. (2017). Zooplankton community structure of the sea farming in Haizhou Bay and its relationships with environment factors. J. Environ. Sci..

[33] Jiang H.C., Chen H.G., Song X.K., Liu N., He J.L., Cheng L., Wang Y.X. (2015). Zooplankton community structure in Jincheng area of Laizhou Bay and its relationship with environmental factors. Acta Ecol. Sin..

[34] Pinto-Coelho R., Pinel-Alloul B., Méthot G. (2005). Crustacean Zooplankton in Lakes and Reservoirs of Temperate and Tropical Regions: Variation with Trophic Status. Can. J. Fish. Aquat. Sci..

[35] Hamil S., Bouchelouche D., Arab S., Alili M., Baha M., Arab A. (2021). The relationship between zooplankton community and environmental factors of Ghrib Dam in Algeria. Environ. Sci. Pollut. Res..

[36] Bișinicu E., Boicenco L., Pantea E., Timofte F., Lazăr L., Vlas O. (2024). Qualitative Model of the Causal Interactions between Phytoplankton, Zooplankton, and Environmental Factors in the Romanian Black Sea. Phycology.

[37] Harvey M., Therriault J.C., Simard N. (2001). Hydrodynamic control of late summer species composition and abundance of zooplankton in Hudson Bay and Hudson Strait (Canada). J. Plankton Res..

[38] Nicolle A., Hansson L.A., Brodersen J., Nilsson P.A., Brönmark C. (2011). Interactions between predation and resources shape zooplankton population dynamics. PLoS ONE.

[39] Fu M., Lin J., Zhang P., Luo W., Zhang J. (2023). Tide drives nutrients variation and exchange flux in the semi-enclosed Shuidong Bay coastal water in winter, South China Sea. Ocean Coast. Manag..

[40] Wu K., Xiu B., Cui D., Lu D., Yang B., Liang S., Zhou J., Huang H., Peng S. (2024). Composition and distribution of nutrients and environmental capacity in Dapeng Bay, northern South China Sea. Mar. Pollut. Bull..

[41] Liu L., Li Y., Sun P., Wang Z.L., Xin M. (2020). Seasonal changes of phytoplankton community structure and its influencing factors in Qinzhou Bay. Mar. Environ. Sci..

[42] Fu T.T., Chen B.H., Ji W.D., Chen H.Z., Chen W.F., Dong X., Kuang W., Wang J., Lin H. (2016). Size structure of phytoplankton community and its response to environmental factors in Xiamen Bay, China. Environ. Earth Sci..

[43] Chen D.D., Pang Q.Z., Chen X.H., Sun P.Y., Tu Z.G. (2020). Community structure of phytoplankton and its relationship with environmental actors in Houshui Bay, Hainan, in spring and autumn, 2018. Trans. Oceanol. Limnol..

[44] Morel F.M.M., Cox E.H., Kraepiel A.M.L., Lane T.W., Milligan A.J., Schaperdoth I., Reinfelder J.R., Tortell P.D. (2002). Acquisition of inorganic carbon by the marine diatom *Thalassiosira weissflogii*. Funct. Plant Biol..

[45] Paytan A., Mackey K.R.M., Chen Y., Lima I.D., Doney S.C., Mahowald N., Labiosa R., Post A.F. (2009). Toxicity of atmospheric aerosols on marine phytoplankton. Proc. Natl. Acad. Sci. USA.

[46] Stuart R.K., Dupont C.L., Johnson D.A., Paulsen I.T., Palenik B. (2009). Coastal strains of marine *Synechococcus* species exhibit increased tolerance to copper shock and a distinctive transcriptional response relative to those of openocean strains. Appl. Environ. Microbiol..

[47] Zhao Z.L., Li H.J., Sun Y., Yang Q., Fan J.F. (2021). Contrasting the Assembly of Phytoplankton and Zooplankton Communities in a Polluted Semi-Closed Sea: Effects of Marine Compartments and Environmental Selection. Environ. Pollut..

[48] Lu M.Q., Luo X., Jiao J.J., Li H.L., Wang X.J., Gao J.Y., Zhang X., Xiao K. (2019). Nutrients and heavy metals mediate the distribution of microbial community in the marine sediments of the Bohai Sea, China. Environ. Pollut..

[49] Jisr N., Younes G., El Omari K., Hamze M., Sukhn C., El-Dakdouki M.H. (2020). Levels of heavy metals, total petroleum hydrocarbons, and microbial load in commercially valuable fish from the marine area of Tripoli, Lebanon. Environ. Monit. Assess..

[50] Yang T.J., Chen Y., Zhou S.Q., Li H.W. (2019). Impacts of aerosol copper on marine phytoplankton: A review. Atmosphere.

[51] Oshida P.S., Word L.S., Mearns A.J. (1981). Effects of hexavalent and trivalent chromium on the reproduction of *Neanthes arenaceodentata* (Polychaeta). Mar. Environ. Res..

[52] Wallen D.G. (1996). Adaptation of the growth of the diatom *Fragilaria crotonensis* (Kitton) and the phytoplankton assemblage of Lake Erie to chromium toxicity. J. Great Lakes Res..

[53] Ali N.A., Dewez D., Didur O., Popovic R. (2006). Inhibition of photosystem II photochemistry by Cr is caused by the alteration of both D1 protein and oxygen evolving complex. Photosynth. Res..

[54] Ramirez-Diaz M.I., Diaz-Perez C., Vargas E., Riveros-Rosas H., Campos-García J., Cervantes C. (2008). Mechanisms of bacterial resistance to chromium compounds. BioMetals.

[55] Sathicq M.B., Gomez N. (2018). Effects of hexavalent chromium on phytoplankton and bacterioplankton of the Rio de la Plata Estuary: An ex-situ assay. Environ. Monit. Assess..

[56] Herman P.H.J., Middelburg J.J., Van De Koppel J., Heip C. (1999). Ecology of estuarine macrobenthos. Adv. Ecol. Res..

[57] Bilyard G.R. (1987). The value of benthic infauna in marine pollution monitoring studies. Mar. Pollut. Bull..

[58] Borja A., Franco J., Perez V. (2000). A Marine Biotic Index to Establish the Ecological Quality of Soft-Bottom Benthos within European Estuarine and Coastal Environments. Mar. Pollut. Bull..

[59] Chapman P.M., Wang F. (2001). Assessing sediment contamination in estuaries. Environ. Toxicol. Chem..

[60] Zhou L.M., Sun Y., Zhang H.H., Yang G.P. (2018). Distribution and characteristics of inorganic nutrients in the surface microlayer and subsurface water of the Bohai and Yellow Seas. Cont. Shelf Res..

[61] Ni D.P., Zhang Z., Liu X.S. (2012). The suitability of AMBI to benthic quality assessment on the intertidal zones of Bohai Sea Benthic ecological quality assessment of the Bohai Sea, China using marine biotic indices. Mar. Pollut. Bull..

[62] Cai W.Q., Meng W., Zhu Y., Zhou J., Liu L.S. (2012). Assessing benthic ecological status in stressed Liaodong Bay (China) with AMBI and M-AMBI. Chin. J. Oceanol. Limnol..

[63] Zhou H., Zhang Z.N., Liu X.S., Hua E. (2012). Decadal change in sublittoral macrofaunal biodiversity in the Bohai Sea, China. Mar. Pollut. Bull..

[64] Jessen C., Voolstra C.R., Wild C. (2014). In situ effects of simulated overfishing and eutrophication on settlement of benthic coral reef invertebrates in the Central Red Sea. PeerJ.

[65] Nijssen M.E., WallisDeVries M.F., Siepel H. (2017). Pathways for the effects of increased nitrogen deposition on fauna. Biol. Conserv..

[66] Nair V.D. (2014). Soil phosphorus saturation ratio for risk assessment in land use systems. Front. Environ. Sci..

[67] Wang Z., Qu F.Y., Sui J.X., Wang Z. (2016). Community structure and diversity of macrobenthos in the western waters of Liaodong Bay during summer. Mar. Sci..

[68] Sawall Y., Richter C., Ramette A. (2012). Effects of Eutrophication, Seasonality and Macrofouling on the Diversity of Bacterial Biofilms in Equatorial Coral Reefs. PLoS ONE.

[69] Li J., Zheng W., Cai Z., Ma J., Li G., Ma B., Zhao J., Li Z., Li S., Chen M. (2024). Changes in the Characteristics of Zooplankton Communities in Response to Shifts in the Aquatic Environment in the Shallow Waters of Northern Liaodong Bay, China. Water.

[70] Wan X.H., Fang Y., Jiang Y., Lu X., Zhu L., Feng J. (2024). Temperature and nutrients alter the relative importance of stochastic and deterministic processes in the coastal macroinvertebrates biodiversity assembly on long-time scales. Ecol. Evol..

[71] Wang K., Zhao L., Zhu Y., Yang L., Wang Y., Hong X. (2024). Characterization of Nutrients, Heavy Metals, Petroleum and Their Impact on Phytoplankton in Laizhou Bay: Implications for Environmental Management and Monitoring. Ocean Coast. Sea Res..

[72] Zhang X.J., Ding L., Feng C.H. (2016). Macrobenthos community and environmental factors in the middle waters of Liao dong Bay in spring. Mar. Sci..

[73] Zou J.Y., Wang H.X., Shan X.J., Gorfine H., Ren Y.P. (2025). Spatial variation of functional structure of fish communities in the Bohai Sea. Glob. Ecol. Conserv..

[74] Ndatimana G., Nantege D., Arimoro F.O. (2023). A review of the application of the macroinvertebrate-based multimetric indices (MMIs) for water quality monitoring in lakes. Environ. Sci. Pollut. Res..

[75] Shi Y., Zhang G., Zhang G.D., Wen Y., Guo Y., Peng L., Xu W., Sun J. (2022). Species and functional diversity of marine macrobenthic community and benthic habitat quality assessment in semi-enclosed waters upon recovering from eutrophication, Bohai Bay, China. Mar. Pollut. Bull..

[76] Zhang J.J., Wang Y.J., Li F., Liu K., Wang Y., Yu Y., Gao Y.J., Xiao X.T., Lü Z.B. (2022). Effects of pollution control of Xiaoqing River on environmental factors and phytoplankton community in the Laizhou Bay. Environ. Sci..

[77] Jiang S., Wang J., Fan W., Chen L., Chen J., Li B. (2024). Decadal variation and temporal stability of the macrobenthic community in the Bohai Sea, China. Mar. Pollut. Bull..

[78] Hooper D.U., Chapin F.S., Ewel J.J., Hector A., Inchausti P., Lavore S., Lawton J.H., Lodge D.M., Loreau M., Naeem S. (2005). Effects of biodiversity on ecosystem functioning: A consensus of current knowledge. Ecol. Monogr..

[79] Gallardo B., Clavero M., Sánchez M.I., Vilà M. (2016). Global ecological impacts of invasive species in aquatic ecosystems. Glob. Change Biol..

[80] Pyšek P., Hulme P.E., Simberloff D., Richardson D.M. (2020). Scientists’ warning on invasive alien species. Biol. Rev..

[81] Gotama R., Baker D.M., Guibert I., McIlroy S.E., Russell B.D. (2024). How a coastal megacity affects marine biodiversity and ecosystem function: Impacts of reduced water quality and other anthropogenic stressors. Ecol. Indic..

[82] Duffy J.E., Godwin C.M., Cardinale B.J. (2017). Biodiversity effects in the wild are common and as strong as key drivers of productivity. Nature.

[83] Benkwitt C.E., Wilson S.K., Graham N.A.J. (2020). Biodiversity increases ecosystem functions despite multiple stressors on coral reefs. Nat. Ecol. Evol..

[84] Moi D.A., Lansac-Tôha F.M., Romero G.Q., Souza T.S., Cardinale B.J., Kratina P., Perkins D.M., Mello F., Jeppesen E., Heino J. (2022). Human pressure drives biodiversity-multifunctionality relationships in large Neotropical wetlands. Nat. Ecol. Evol..

[85] Naeem S., Li S. (1997). Biodiversity enhances ecosystem reliability. Nature.

[86] Cardinale B., Duffy J., Gonzalez A., Hooper D., Perrings C., Venail P., Narwani A., Mace G., Tilman D., Wardle D. (2012). Biodiversity loss and its impact on humanity. Nature.

[87] Yachi S., Loreau M. (1999). Biodiversity and ecosystem productivity in a fluctuating environment: The insurance hypothesis. Proc. Natl. Acad. Sci. USA.

[88] Sutherland W.J., Pullin A.S., Dolman P.M., Knight T.M. (2004). The need for evidence-based conservation. Trends Ecol. Evol..

